# Supplementary material for: Cr-spinel records metasomatism not petrogenesis of mantle rocks
Source: Nat Commun. 2019 Nov 8;10:5103. doi: 10.1038/s41467-019-13117-1 (PMC6841941; doi:10.1038/s41467-019-13117-1)
Supplement: Supplementary file 1 — Supplementary Information [file 41467_2019_13117_MOESM1_ESM.docx]

**Supplement information for:**

**Cr-spinel records metasomatism not petrogenesis of mantle rocks**

**By Gamal El Dien et al.**

**Supplementary Figures.**


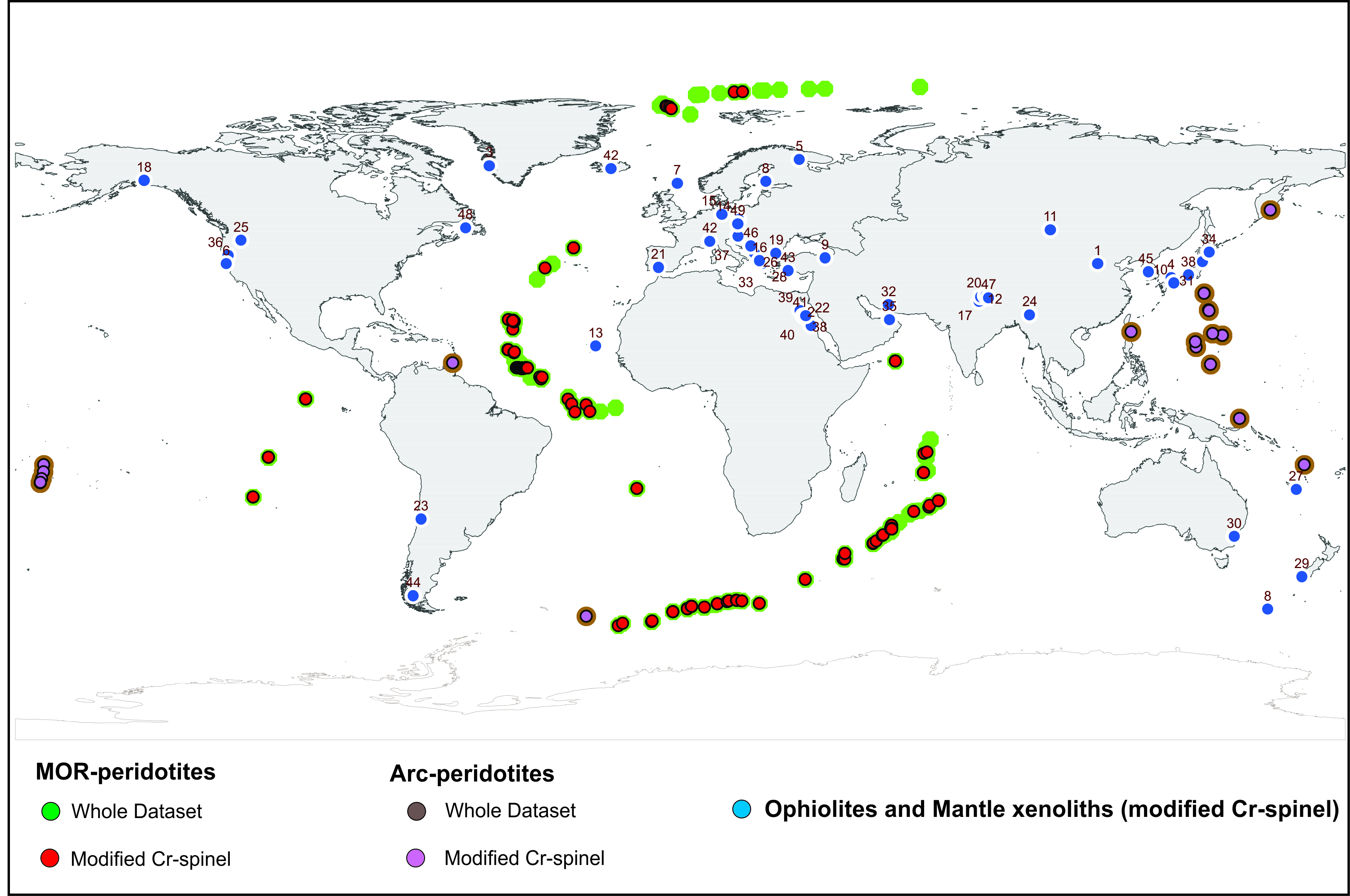


**Supplementary Figure 1: Samples location of perdiotite rocks that have modified spinel.** The map shows the distribution of mid-ocean ridge (MOR) and arc-peridotites whole dataset used in this study and ones that have Al-Cr reverse zoning in spinel and modified spinel that passed our filter. In addition to some examples of on-land ophiolites and mantle xenolthis that also have modified Cr-spinel. Only a small proportion of the original authors clearly stated spinel heterogeneity. The on-land ophiolites samples have ages ranging from Archean to the Present. See **Supplementary Data**1 for more details about this record. Our compilation is certainly not an exhaustive one of all results on modified spinel as such features have commonly been ignored in previous studies, but it does illustrate how widespread such a feature is, and forms a base for future data addition.


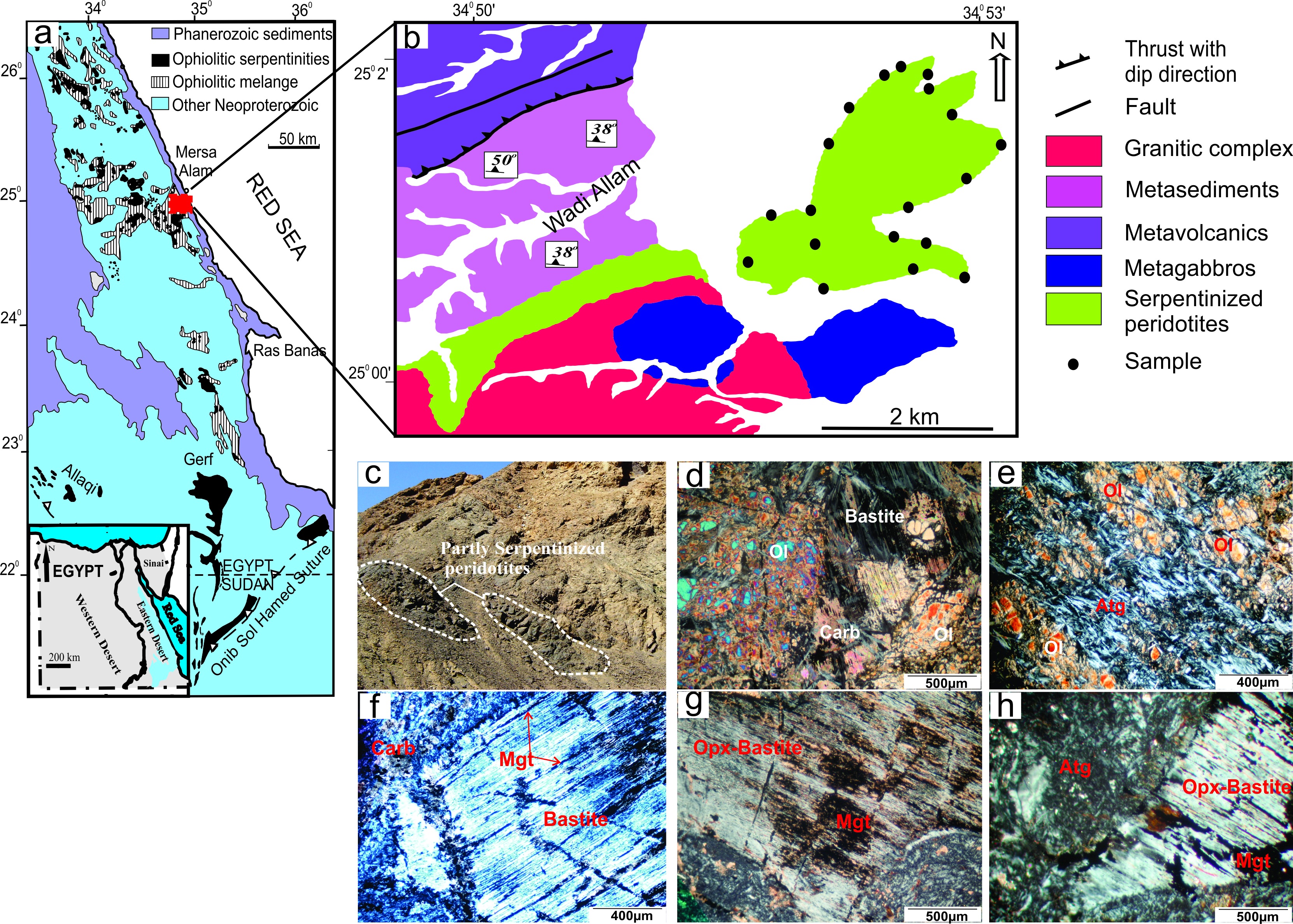


**Supplementary Figure 2: Simplified geological maps of the Eastern Desert region in Egypt and study area with sampling locations.** (a) Distribution of ophiolitic rocks in the Eastern Desert of Egypt^1^. (b) Geological map of the Wadi Alam serpentinized peridotites^2^. (c) Partially serpentinized peridotites showing lensoidal bodies. (d) Olivine (Ol) relics forming mesh texture and bastite texture, with oriented magnetite grains along cleave planes (e) Olivine cracked crystals dissected by antigorite (Atg) serpentine network. (f-h) Orthopyroxene (Opx) bastite texture has small magnetite (Mgt) grains distributed along original pyroxene cleavage. Carb = Carbonates.


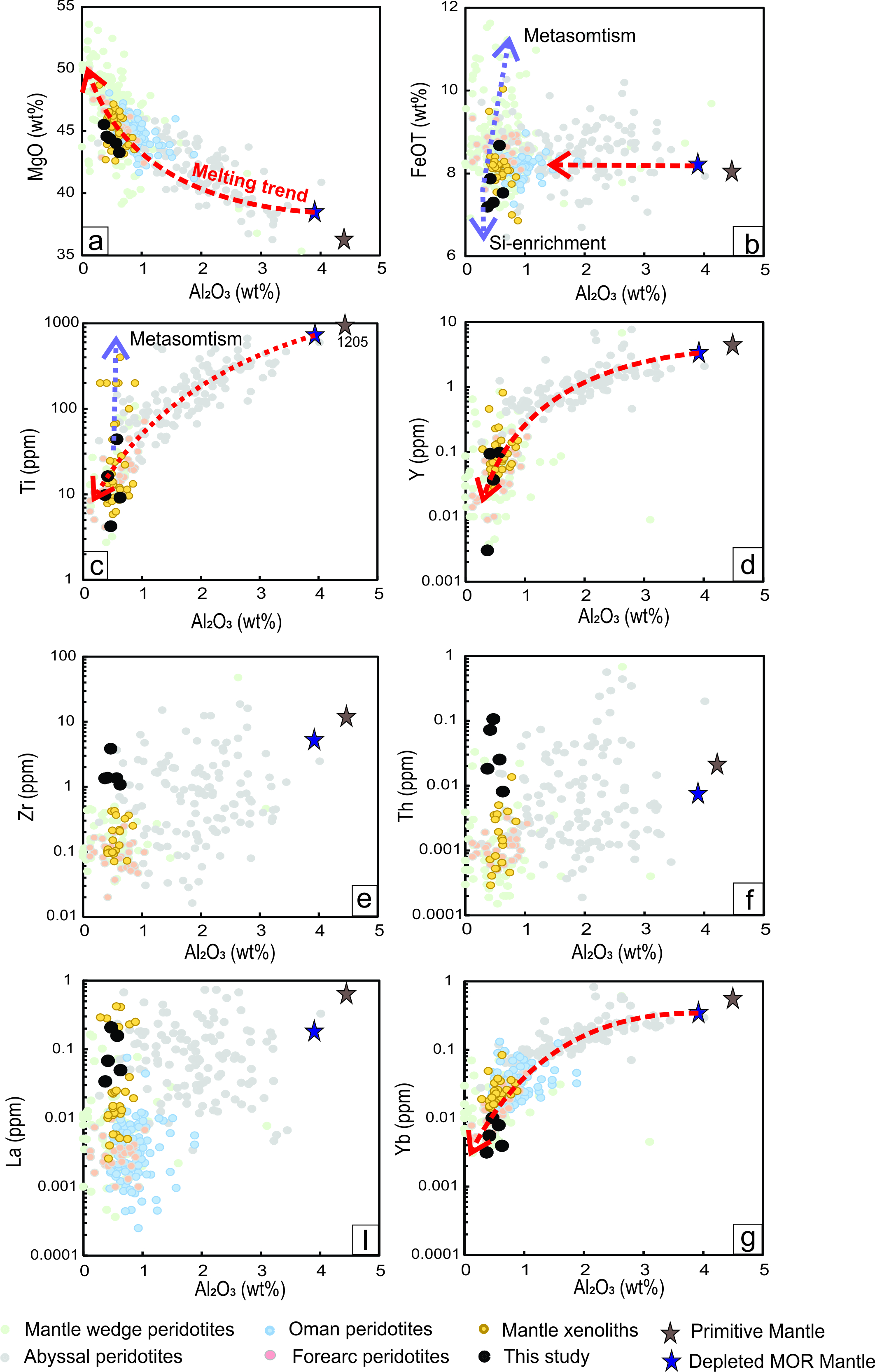


**Supplementary Figure 3: Whole-rock Al_2_O_3_ (wt %) vs. selected major, trace and rare earth elements variation of the studied peridotites.** Fields of abyssal peridotites^3,4^, forearc peridotites^5^, Oman peridotites^6^, mantle wedge peridotites^3^ and mantle xenoliths^7-10^. Composition of primitive mantle^11^ and depleted MORB mantle (DMM)^12^ is plotted for comparison.


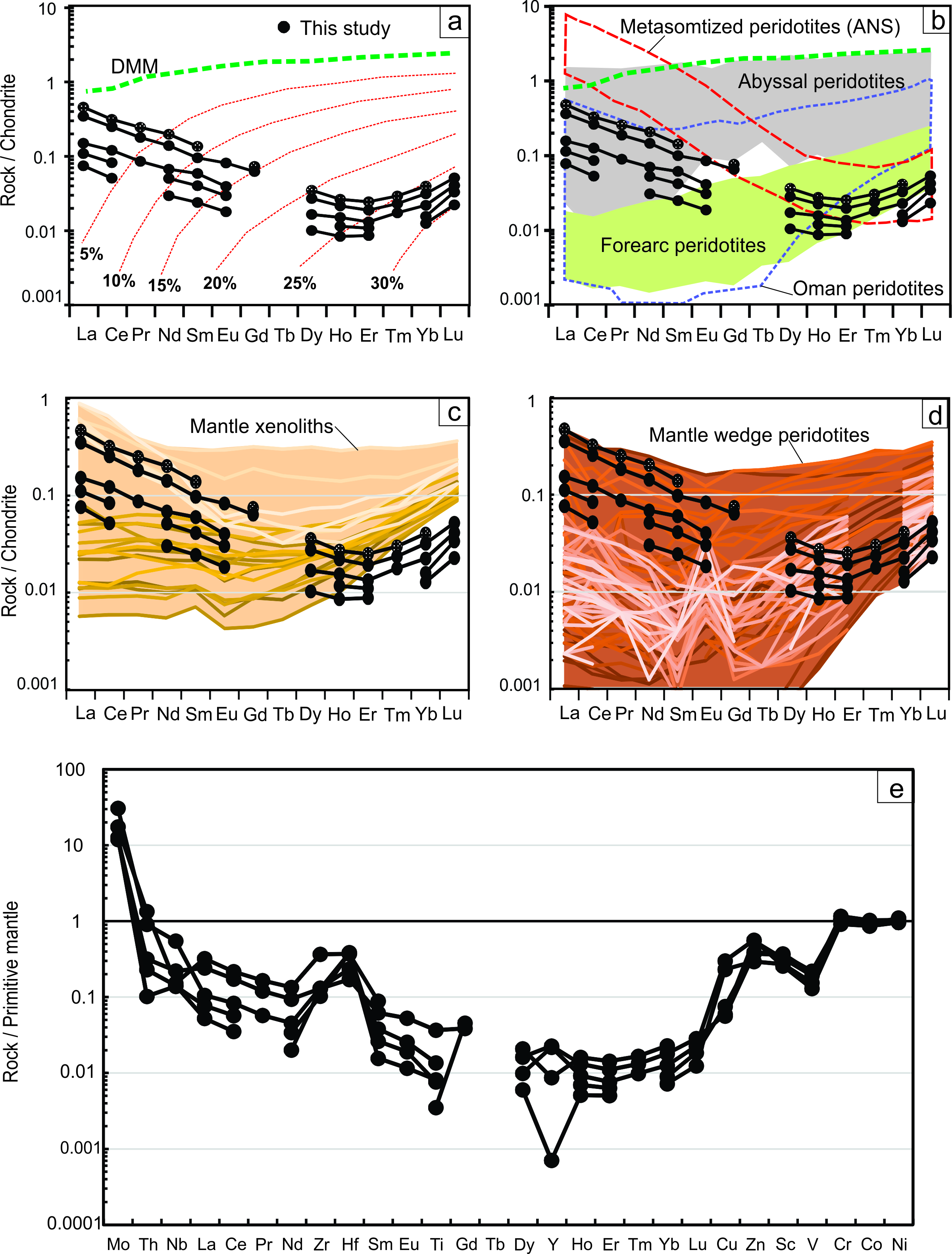


**Supplementary Figure 4: Primitive mantle-normalized**^11^ **multielement and rare earth element patterns normalized to chondrite**^13^ **of the studied peridotites.** (a) Estimation of degree of partial melting for the studied rocks by using the non-modal fractional melting model of a source of depleted MORB mantle (DMM)^12^, with melting curves based on an assumed source of DMM composition in the spinel stability field^14^. (b-c) A comparison our studied rocks with that of different tectonic settings and metasomatized peridotites from Arabian-Nubian Shield (ANS; after Hamdy et al.^15^). (e) Primitive mantle normalized elements for the studied rock showing a gradual increase from rare earth elements to high field strength elements and high enrichment in Mo.


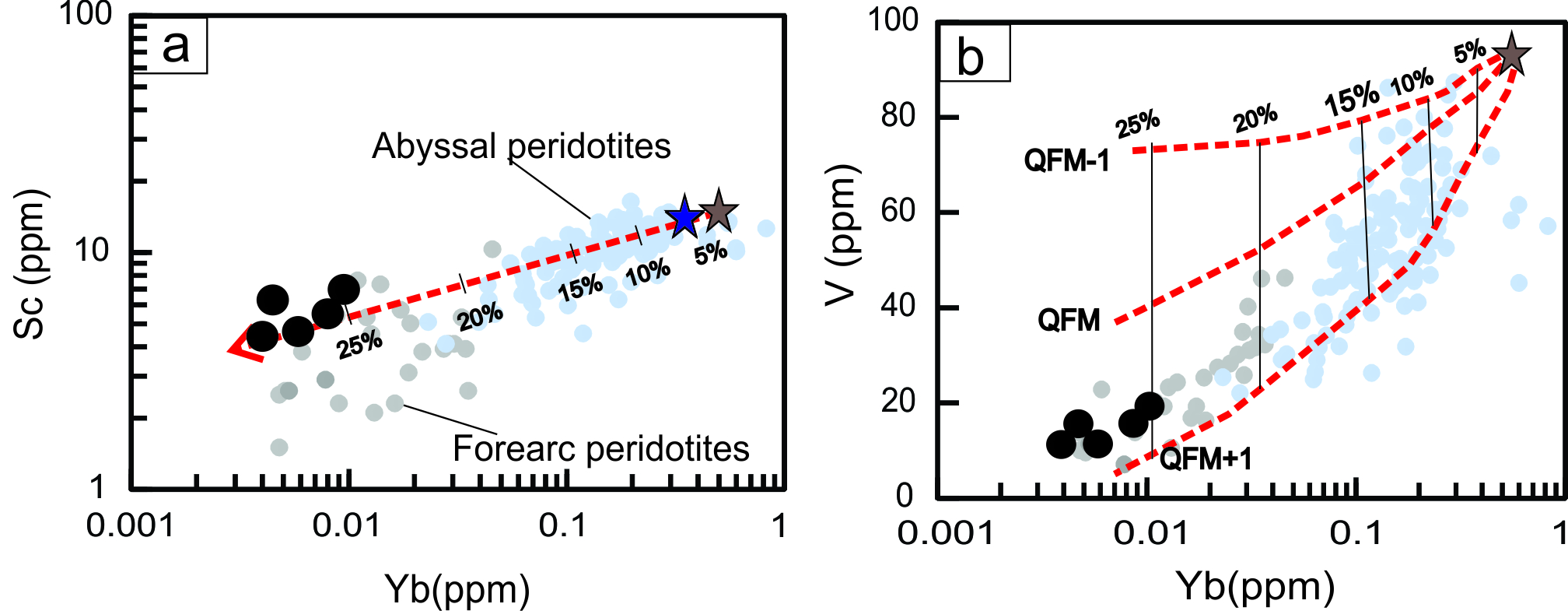


**Supplementary Figure 5: A determination of the degree of partial melting of the studied rocks based on bulk-rock chemistry.** The plots of Yb (ppm) vs. Sc ppm (a) and V ppm (b) are for residual peridotites according to Lee et al.^16^, with annotated degrees of mantle melting (in %). Diagrams contain fractional melting trends for different oxygen fugacities. FMM = Fertile MORB mantle. Vanadium behaves as a moderately incompatible element while melting under reducing conditions (QFM–1; this refers to log ƒO_2_ (QFM) = log units relative to quartz-fayalite-magnetite buffer), resulting in low depletion of V; under oxidizing conditions (QFM+1), the ratio of V^3+^/ (V^4+^+V^5+^) is low and partition coefficients are high. Thus vanadium as a highly incompatible element is readily depleted in the mantle during partial melting. Legends for symbols are the as in Fig. S3


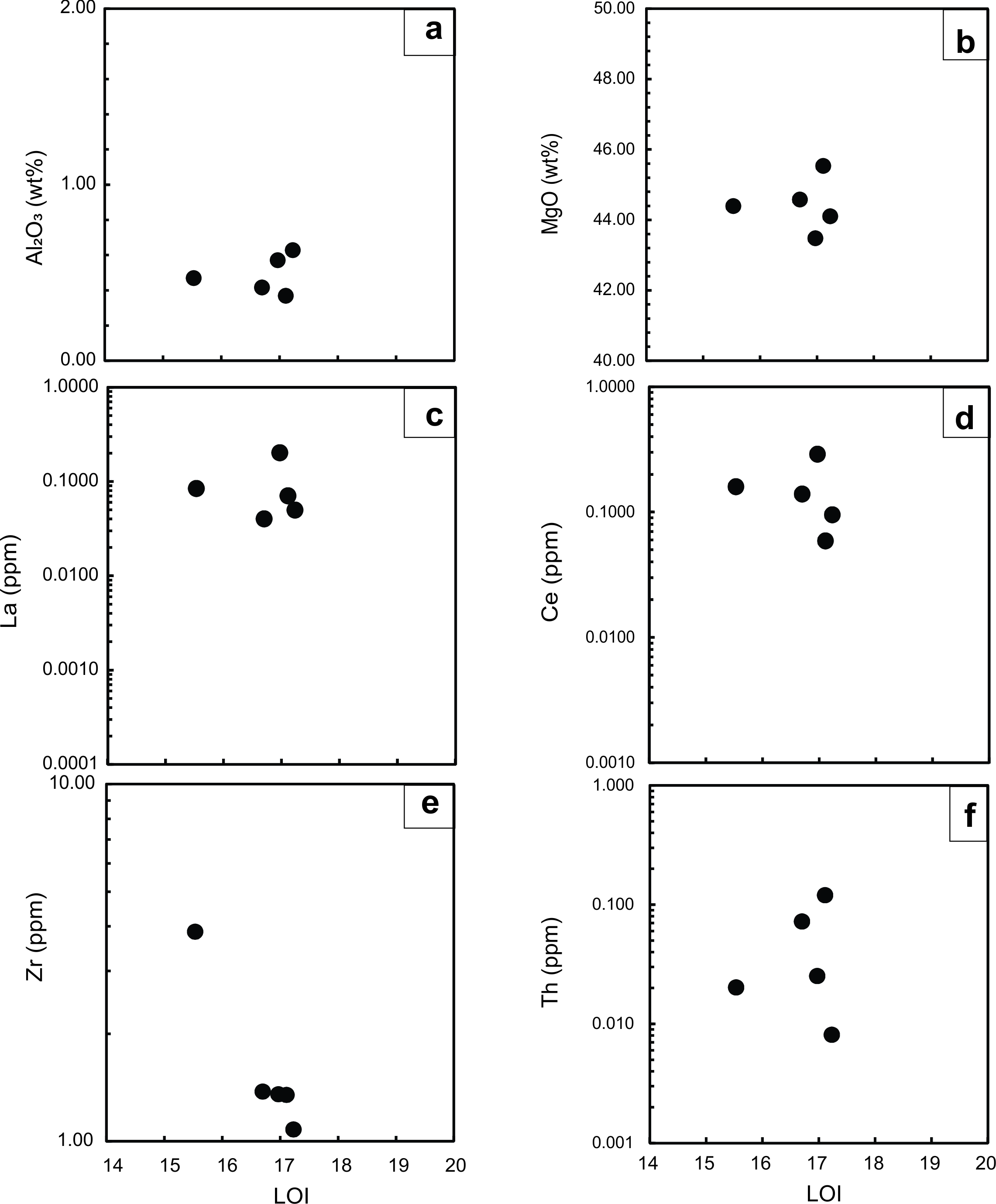


**Supplementary Figure 6: Variation diagrams of LOI (wt%) vs. selected major, trace and rare earth elements of bulk composition of the studied peridotites.** The plots show no correlation between LOI and the selected elements.


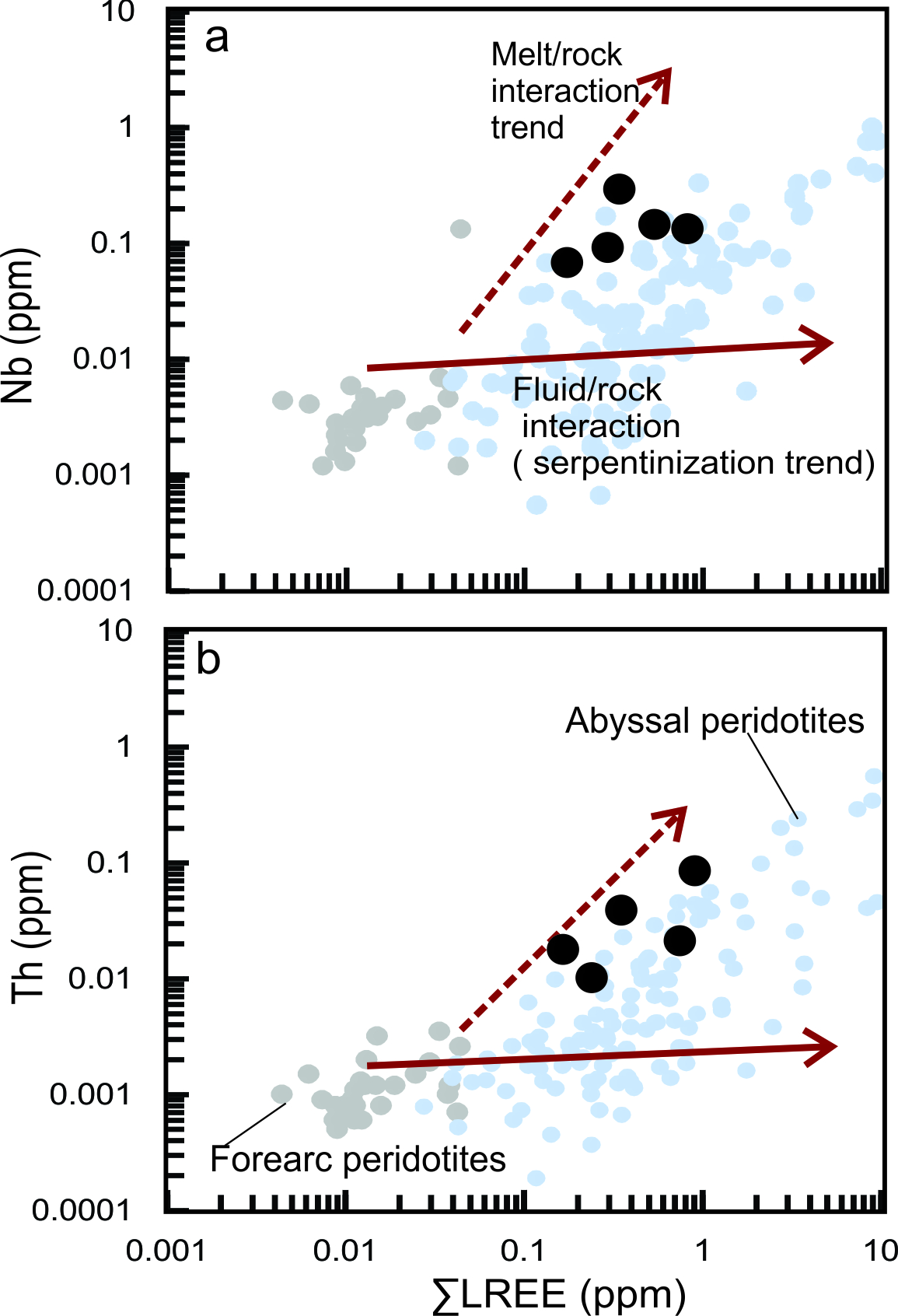


**Supplementary Figure 7: Co-variation of HFSE and LREE.** (a) Nb vs ƩLREE and (b) Th vs. ƩLREE. The plots show the melt-rock interaction and fluid-rock interaction ( serpentinization-related) trends^17^. The studied perdioites have beed affect by post melting melt/rock interactions process that led to HFSE enrichements. Legends for symbols are the as in Fig. S3


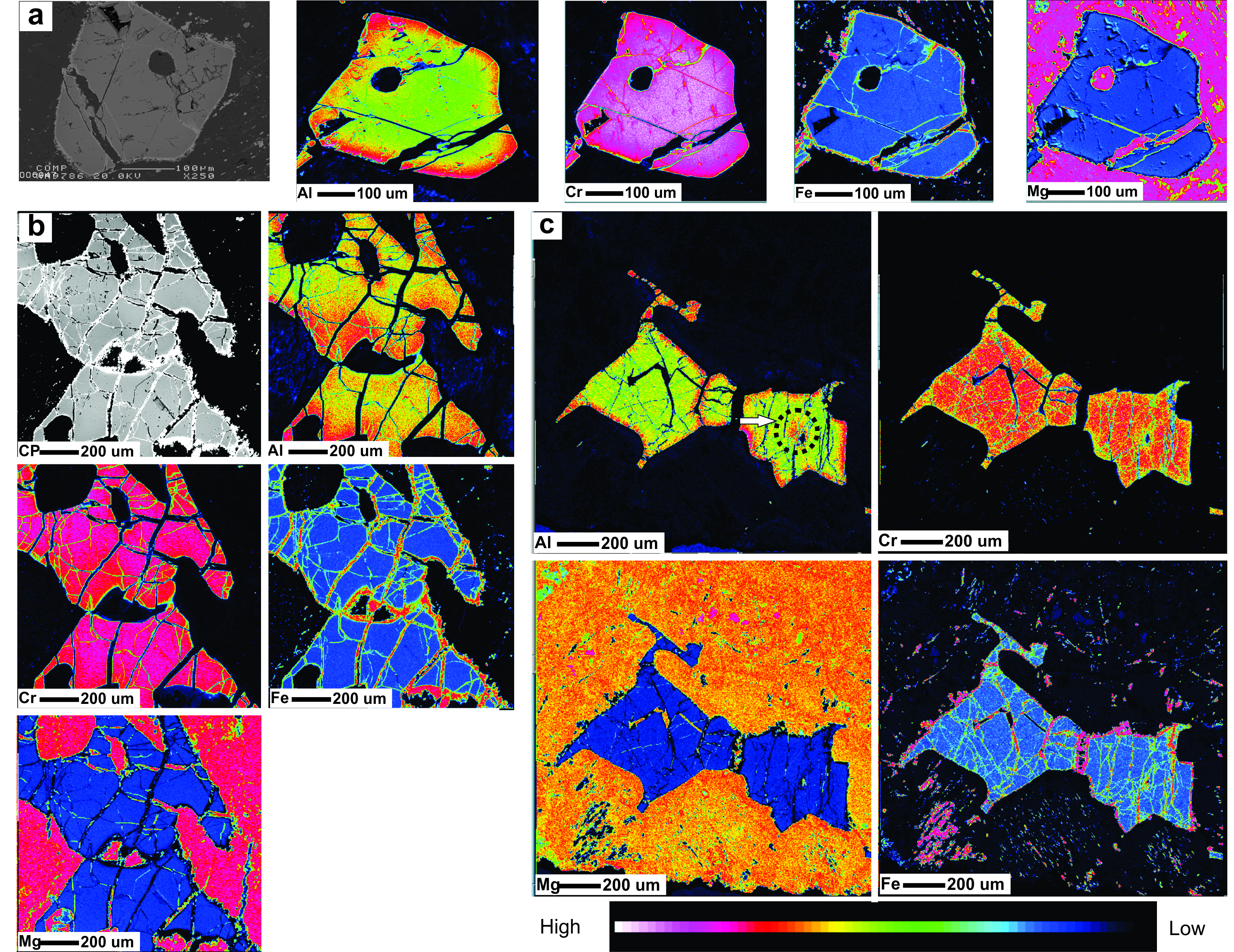


**Supplementary Figure 8: Al-Cr-Fe-Mg X-ray elemental maps for the studied Cr-Spinel.** The maps show heterogeneity in Al-Cr content distribution within the grains, but relatively homogenous Fe and Mg distribution between the cores and the rims.


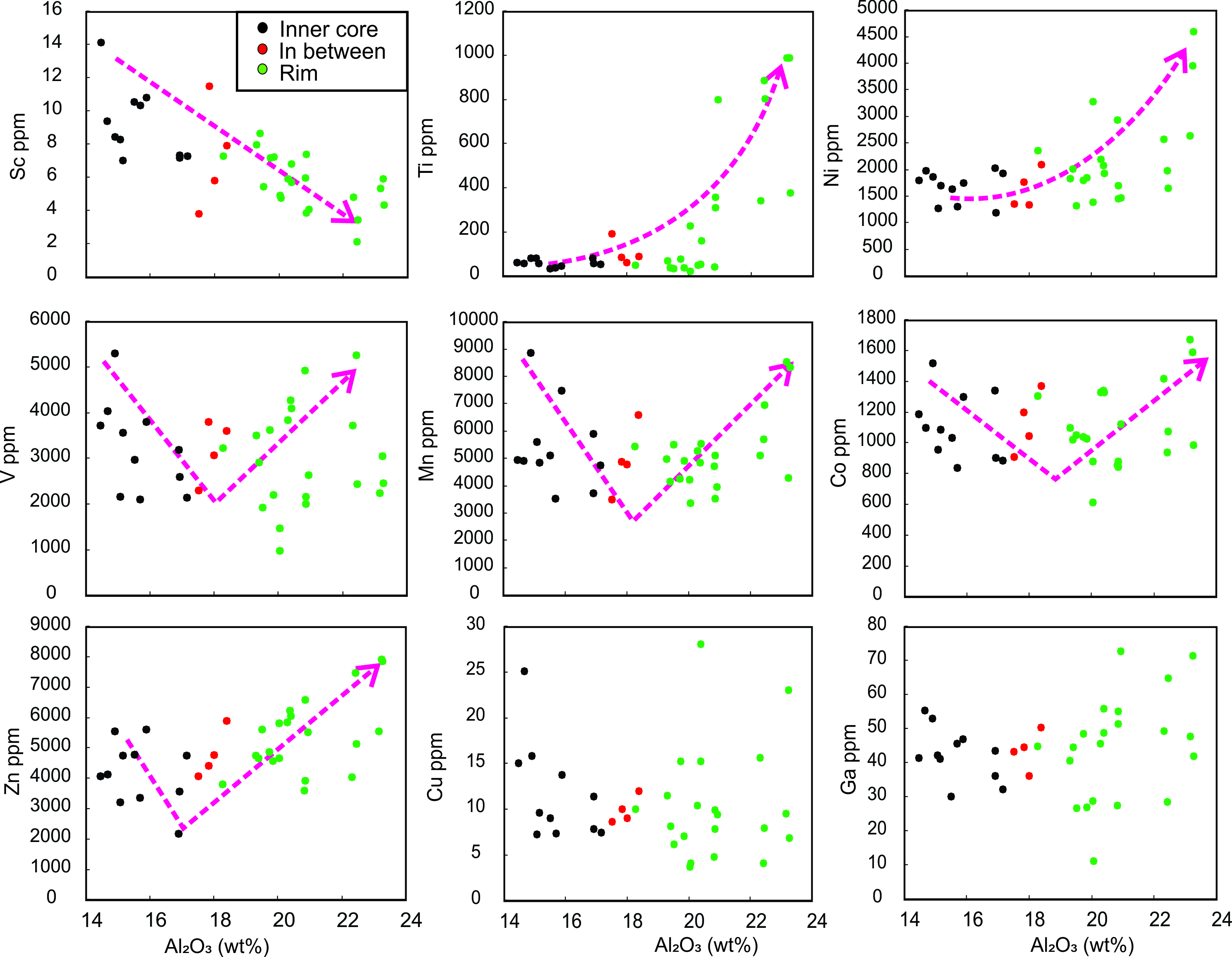


**Supplementary Figure 9: Binary plots of Al_2_O_3_ (wt %) vs. transitions elements for the studied Cr-spinel.** Sc shows negative relation, Ti and Ni show positive relation, V, Mn, Co and Zn show a V-shape relation, whereas Cu and Ga show no relation.


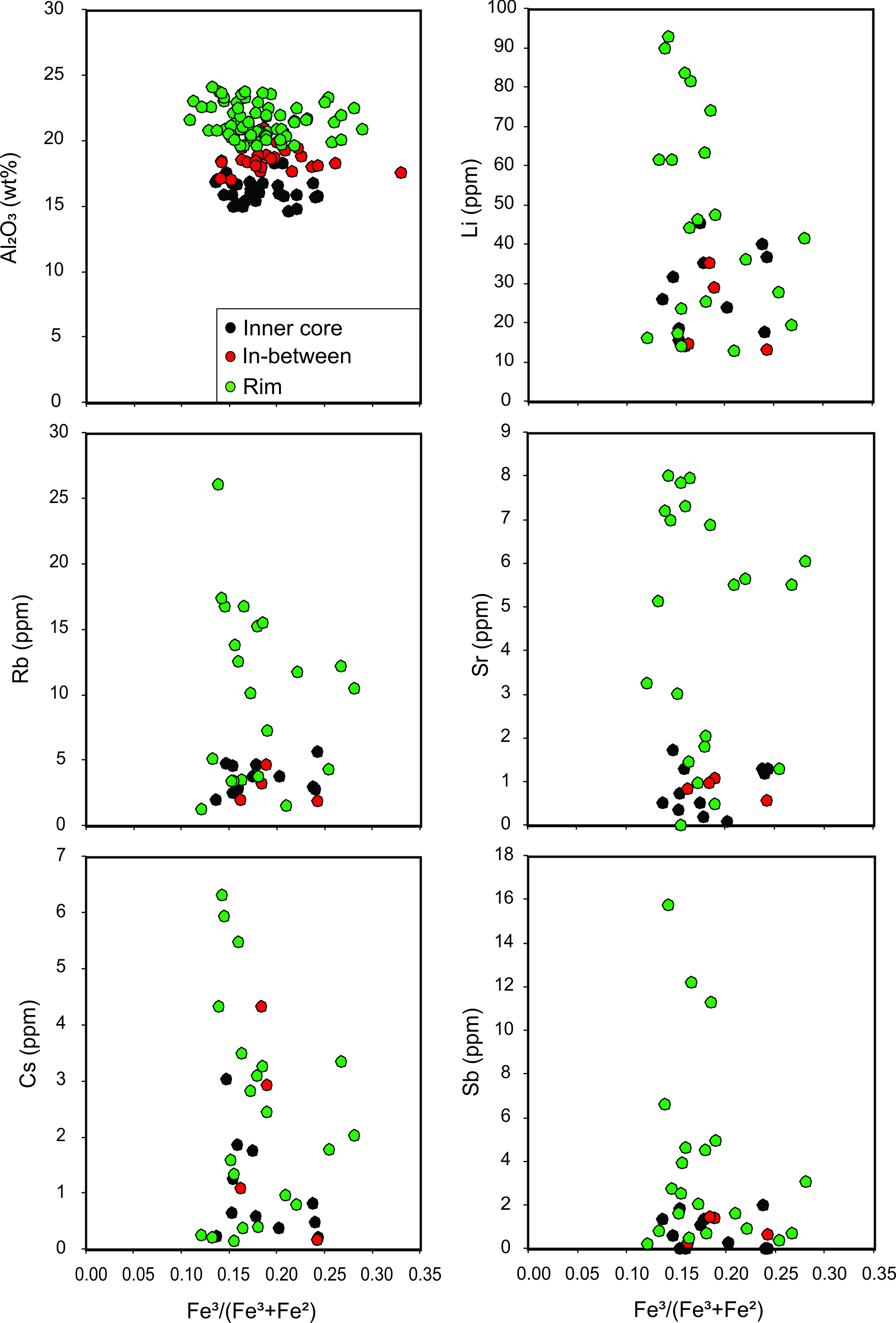


**Supplementary Figure 10: Binary plots of Fe^3+^/(Fe^3+^ + Fe^2+^) ratio vs Al_2_O_3_ (wt %) and FME (ppm) for the studied Cr-spinel.** The plot show that Fe^3+^/(Fe^3+^ + Fe^2+^) ratio has a constant values between the Cr-spinel cores and rims and has no relationship with Al and FME.

**
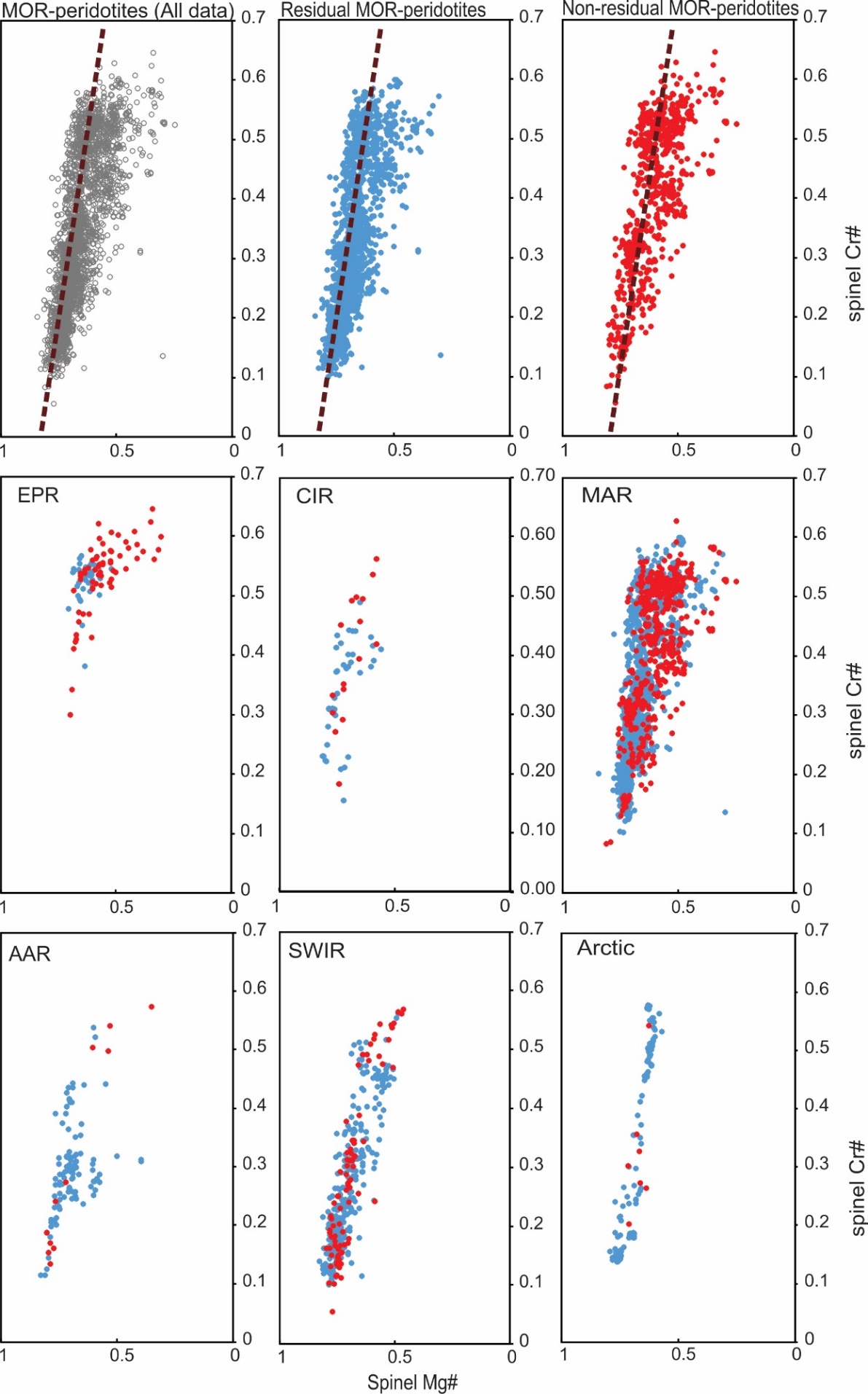
**

**Supplementary Figure 11: Compositional variation for Cr-spinel in the global datasets for abyssal peridotites.** The plots show Cr-spinel Cr# and Mg# in different mid-ocean ridges peridotites and defined as residual and non-residual peridotites. Red dash line represents melting trend from Dick and Bullen^18^. EPR= East Pacific Rise, CIR= Central Indian Ridge, MAR= Mid-Atlantic Ridge, AAR= American-Antarctic Ridge, SWIR= Southwest Indian Ridge and Arctic including Gakkel ridge, Lena trough and Molloy ridge.

**
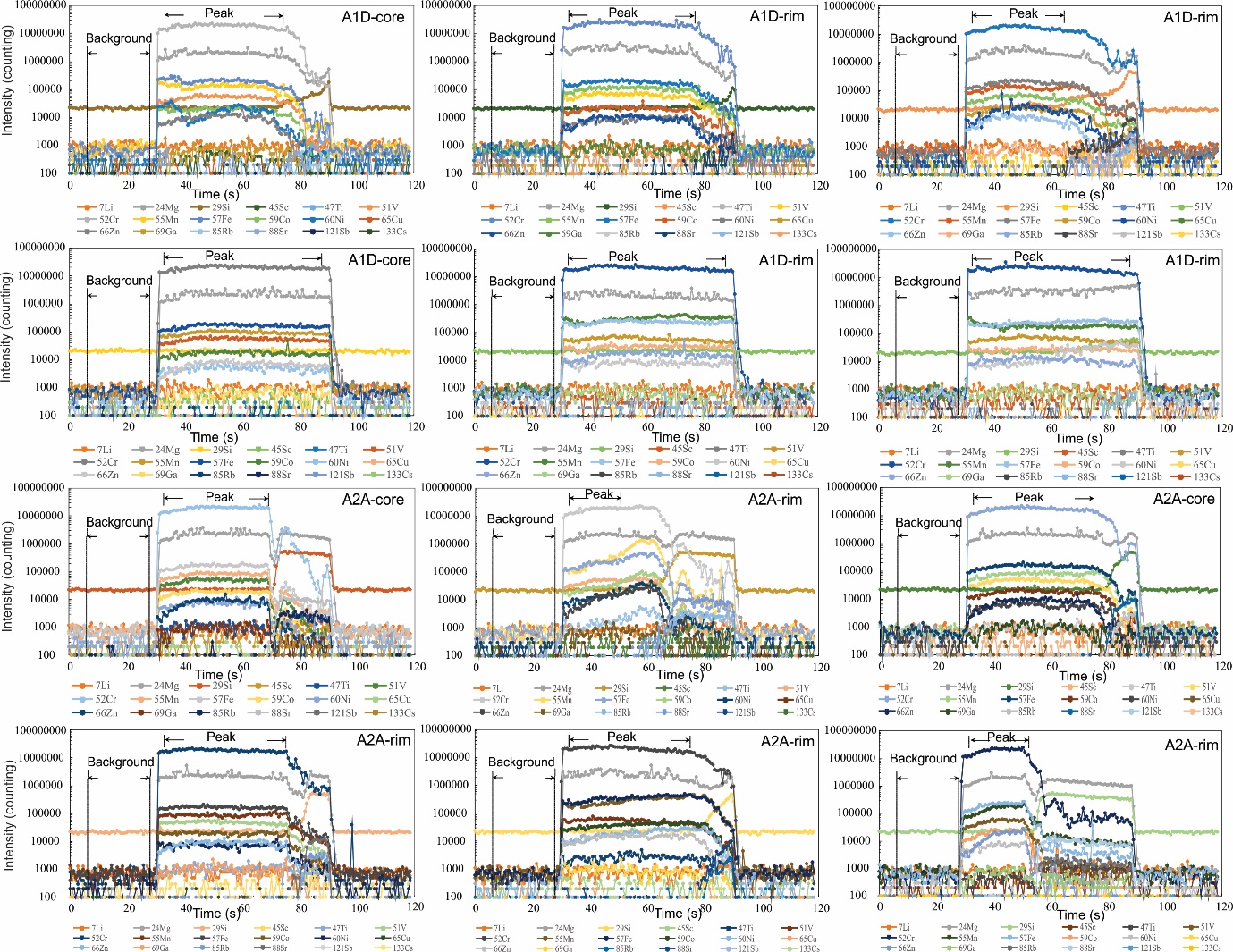
**

**Supplementary Figure 12: LA-ICP-MS ablation profils of the counting vs time (s) for trace element analyses for the studied Cr-spinel.** All the calculated peaks area do not have any significant positive peaks for 29Si and 57Fe which indicated that there are no silicate inclusions and/or magnetite measured.


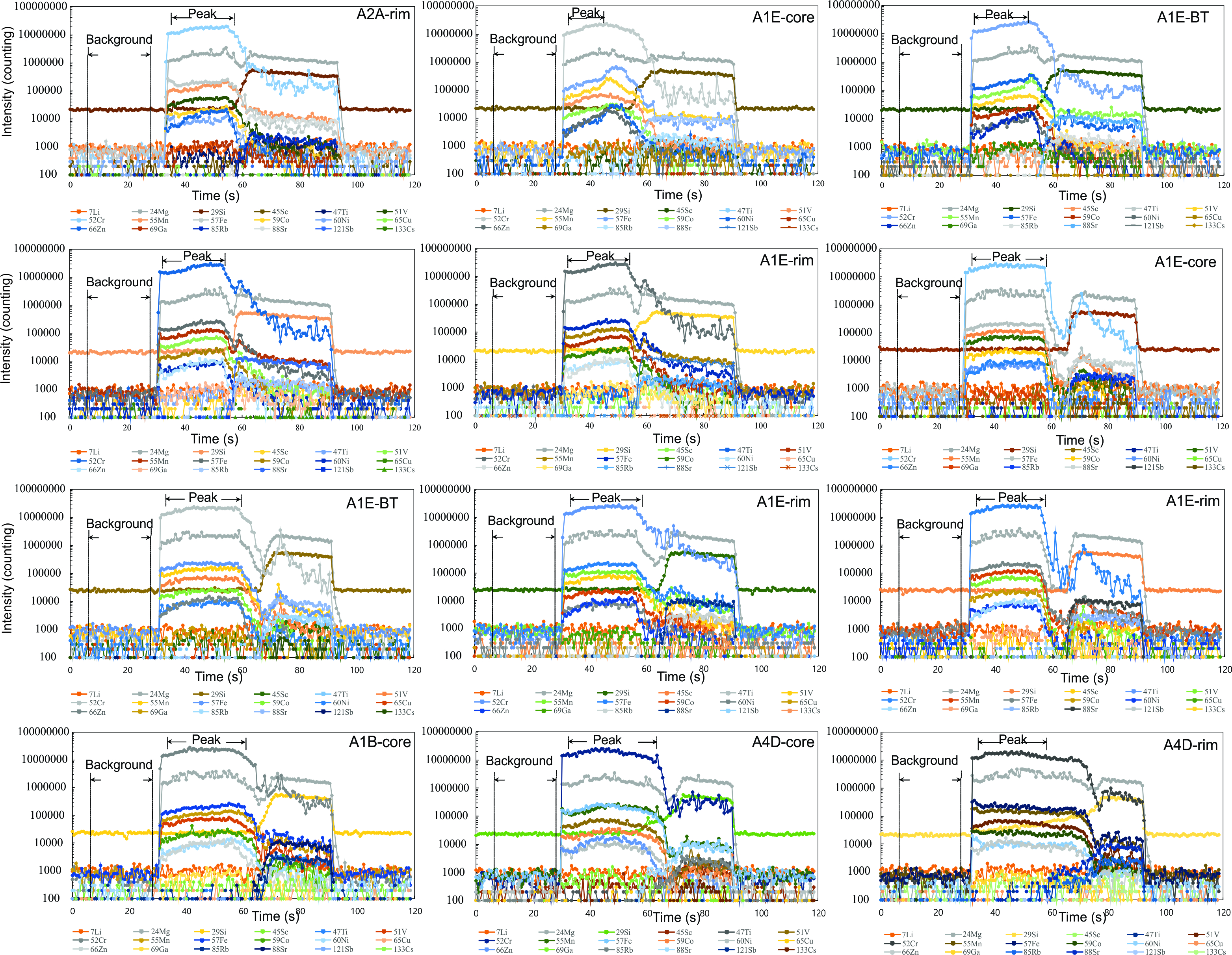


**Supplementary Figure** 12 continue


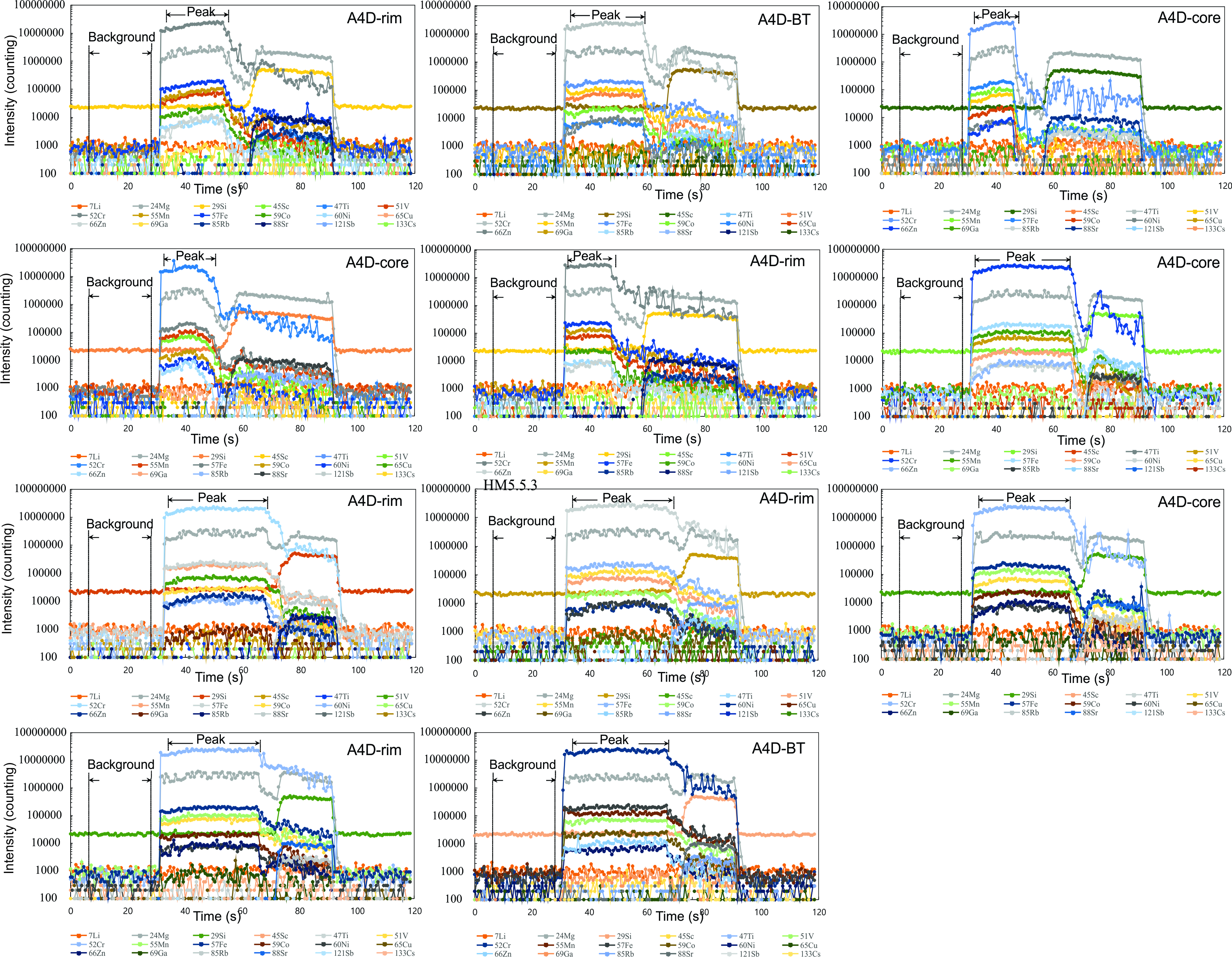


**Supplementary Figure** 12 continue

**
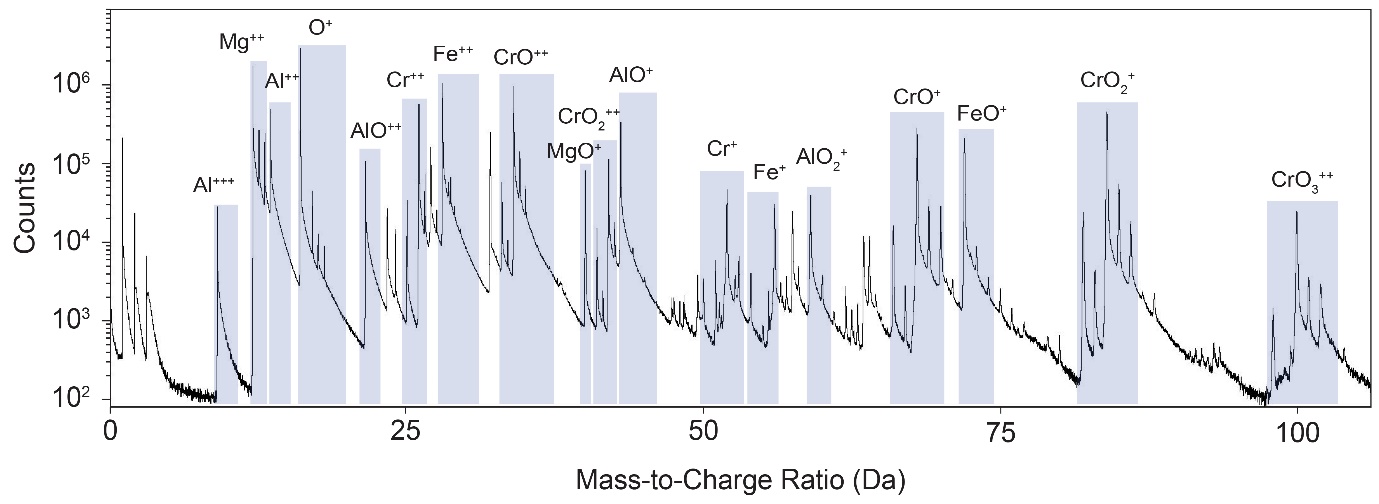
Supplementary Figure 13: Atom probe mass spectrum of the studied Cr-spinel.** The cations are present as different molecular species with singly-charged to triply-charged ions. . For example, Cr is present as Cr^+^, Cr^++^, CrO^+^, CrO^++^, CrO_2_^+^, CrO_2_^++^, CrO_3_^+^ and Cr_2_O_3_^+^; and Al is present as Al^+^, Al^++^, Al^+++^, AlO^+^, AlO^++^, AlO_2_^+^, Al_2_O^++^, and AlCrO_3_^++^.

**Supplementary References**

1. Johnson, P. R. *et al.* Late Cryogenian-Ediacaran history of the Arabian-Nubian Shield: A review of depositional, plutonic, structural, and tectonic events in the closing stages of the northern East African Orogen. *J. African Earth Sci.* **61,** 167–232 (2011).

2. Hamdy, M. M. & Gamal El Dien, H. Nature of serpentinization and carbonation of ophiolitic peridotites (Eastern Desert, Egypt): constrains from stable isotopes and whole-rock geochemistry. *Arab. J. Geosci.* **10,** (2017).

3. Deschamps, F., Godard, M., Guillot, S. & Hattori, K. Geochemistry of subduction zone serpentinites: A review. *Lithos* **178,** 96–127 (2013).

4. Peters, D., Bretscher, A., John, T., Scambelluri, M. & Pettke, T. Fluid-mobile elements in serpentinites: Constraints on serpentinisation environments and element cycling in subduction zones. *Chem. Geol.* **466,** 654–666 (2017).

5. Parkinson, I. J. & Pearce, J. A. Peridotites from the Izu – Bonin – Mariana Forearc (ODP Leg 125): Evidence for Mantle Melting and Melt – Mantle Interaction in a Supra-Subduction Zone Setting. *J. Petrol.* **39,** 1577–1618 (1998).

6. Le Mée, L., Girardeau, J. & Monnier, C. Mantle segmentation along the Oman ophiolite fossil mid-ocean ridge. *Nature* **432,** 167–172 (2004).

7. Franz, L., BECKER, K.-P., KRAMER, W. & HERZIG, A. P. M. Metasomatic Mantle Xenoliths from the Bismarck Microplate (Papua New Guinea)--Thermal Evolution, Geochemistry and Extent of Slab-induced Metasomatism. *J. Petrol.* **43,** 315–343 (2002).

8. Arai, S., Ishimaru, S. & Okrugin, V. M. Metasomatized hazburgite xenoliths from Avacha volcano as fragments of mantle wedge of the Kamchatka arc: Implication for the metasomatic agent. *Isl. Arc* **12,** 233–246 (2003).

9. Ishimaru, S., Arai, S., Ishida, Y., Shirasaka, M. & Okrugin, V. M. Melting and multi-stage metasomatism in the mantle wedge beneath a frontal arc inferred from highly depleted peridotite xenoliths from the avacha volcano, Southern Kamchatka. *J. Petrol.* **48,** 395–433 (2007).

10. Ionov, D. A. Petrology of mantle wedge lithosphere: New data on supra-subduction zone peridotite xenoliths from the andesitic Avacha volcano, Kamchatka. *J. Petrol.* **51,** 327–361 (2010).

11. McDonough, W. . & Sun, S. –. The composition of the Earth. *Chem. Geol.* **120,** 223–252 (1995).

12. Workman, R. K. & Hart, S. R. Major and trace element composition of the depleted MORB mantle (DMM). *Earth Planet. Sci. Lett.* **231,** 53–72 (2005).

13. Anders, E. & Grevesse, N. Abundances of the elements: Meteoritic and solar. *Geochim. Cosmochim. Acta* **53,** 197–214 (1989).

14. De Hoog, J. C. M., Janák, M., Vrabec, M. & Froitzheim, N. Serpentinised peridotites from an ultrahigh-pressure terrane in the Pohorje Mts. (Eastern Alps, Slovenia): Geochemical constraints on petrogenesis and tectonic setting. *Lithos* **109,** 209–222 (2009).

15. Hamdy, M. M., Harraz, H. Z. & Aly, G. A. Pan-African (intraplate and subduction-related?) metasomatism in the Fawakhir ophiolitic serpentinites, Central Eastern Desert of Egypt: mineralogical and geochemical evidences. *Arab. J. Geosci.* **6,** 13–33 (2013).

16. Lee, C.-T. A., Brandon, A. D. & Norman, M. Vanadium in peridotites as a proxy for paleo-fO2 during partial melting: prospects, limitations, and implications. *Geochim. Cosmochim. Acta* **67,** 3045–3064 (2003).

17. Paulick, H. *et al.* Geochemistry of abyssal peridotites (Mid-Atlantic Ridge, 15°20′N, ODP Leg 209): Implications for fluid/rock interaction in slow spreading environments. *Chem. Geol.* **234,** 179–210 (2006).

18. Dick, H. J. B. & Bullen, T. Chromian spinel as a petrogenetic indicator in abyssal and alpine-type peridotites and spatially associated lavas. *Contrib. to Mineral. Petrol.* **86,** 54–76 (1984).
