# Supplementary material for: Cr-spinel records metasomatism not petrogenesis of mantle rocks
Source: Nat Commun. 2019 Nov 8;10:5103. doi: 10.1038/s41467-019-13117-1 (PMC6841941; doi:10.1038/s41467-019-13117-1)
Supplement: Supplementary file 3 — Description of Additional Supplementary Files [file 41467_2019_13117_MOESM3_ESM.docx]

**Description of Supplementary Files**

**File Name: Supplementary Data 1**

**Description:** Summary of ophiolites, mantle xenoliths and MOR-peridotites that have modified Cr-spinel at different scales.

**File Name: Supplementary Data 2**

**Description:** Whole-rock and Cr-spinel geochemical data of the studied rocks.

**File Name: Supplementary Data 3**

**Description:** Cr-spinel database from MOR- and arc-peridotites.

**File Name: Supplementary Movie 1**

**Description:** 3D distribution of Al atoms at sub-nanometer resolution in Cr-spinel rim of specimen number M6.

**File Name: Supplementary Movie 2**

**Description:** 3D distribution of Al atoms at sub-nanometer resolution in Cr-spinel core of specimen number M10.
